# Effect of haemolysis on an enzymatic measurement of ethanol

**DOI:** 10.11613/BM.2021.010704

**Published:** 2020-12-15

**Authors:** Abdulkadir Çat, Kamil Taha Uçar, Alper Gümüş

**Affiliations:** Istanbul Gaziosmanpasa Training and Research Hospital, Medical Biochemistry, Istanbul, Turkey

**Keywords:** ethanol, haemolysis, interference, preanalytical error

## Abstract

**Introduction:**

We investigated the interference of haemolysis on ethanol testing carried out with the Synchron assay kit using an AU680 autoanalyser (Beckman Coulter, Brea, USA).

**Materials and methods:**

Two tubes of plasma samples were collected from 20 volunteers. Mechanical haemolysis was performed in one tube, and no other intervention was performed in the other tube. After centrifugation, haemolysed and non-haemolysed samples were diluted to obtain samples with the desired free haemoglobin (Hb) values (0, 1, 2, 5, 10 g/L). A portion of these samples was then separated, and ethanol was added to the separated sample to obtain a concentration of 86.8 mmol/L ethanol. After that, these samples were diluted with ethanol-free samples with the same Hb concentration to obtain samples containing 43.4, 21.7, and 10.9 mmol/L. Each group was divided into 20 equal parts, and an ethanol test was carried out. The coefficient of variation (CV), bias, and total error (TE) values were calculated.

**Results:**

The TE values of haemolysis-free samples were approximately 2-5%, and the TE values of haemolysed samples were approximately 10-18%. The bias values of haemolysed samples ranged from nearly - 6.2 to - 15.7%.

**Conclusions:**

Haemolysis led to negative interference in all samples. However, based on the 25% allowable total error value specified for ethanol in the Clinical Laboratory Improvement Amendments (CLIA 88) criteria, the TE values did not exceed 25%. Consequently, ethanol concentration can be measured in samples containing free Hb up to 10 g/L.

## Introduction

Ethanol has been consumed widely worldwide throughout history. It has been reported that ethanol is one of the most common substances of abuse (*1*). Ethanol intoxication and ethanol-related injuries constitute an essential burden in emergency departments. A total of 1.2% of all admissions to the emergency department was due to alcohol poisoning (*2*). Although haemolysis is seen in approximately 3% of all samples accepted by the laboratory, the rate of haemolysis samples has been reported to be 6-30% in studies examining samples accepted from the emergency department (*3*, *4*). Therefore, the risk of encountering haemolysis is higher in the samples that are collected for ethanol measurement.

The reference measurement method for the ethanol test is gas chromatography (*5*). However, this method has disadvantages regarding time and cost-effectiveness. For this reason, rapid enzymatic ethanol measurement methods have been developed for use in clinical laboratories. In these procedures, the oxidation of ethanol to acetaldehyde by alcohol dehydrogenase (ADH) and the simultaneous reduction of NAD^+^ to NADH is used to measure the ethanol value. The increase in absorbance at 340 nm is commensurate to the ethanol concentration (*6*).

Haemolysis may interfere with many biochemical tests *via* spectral, chemical, diluent, and additive-induced mechanisms and can cause erroneous results (*7*). On the other hand, some biochemical tests can be measured without significant error, even with high haemolysis values. The accuracy of ethanol testing in haemolysed specimens has been discussed in the literature. In these studies, it has been shown that haemolysis causes a negative bias in enzymatic ethanol measurement (*6*, *8*–*10*). However, the authors have not found a study performed according to the Clinical and Laboratory Standards Institute (CLSI) guidelines. Furthermore, haemolysis interference is dependent on the analytical method and analyser used (*11*). The authors could not access a haemolysis interference study on the ethanol kit they used.

In our study, we aimed to evaluate the interference of haemolysis in ethanol testing carried out with the Synchron assay kit at clinical decision concentrations for ethanol measurement and to compare the results found in the study with the manufacturer’s statements.

## Materials and methods

This study was conducted at the Medical Biochemistry Laboratory of the Gaziosmanpasa Taksim Training and Research Hospital in November 2019 with the approval of the local Ethics Committee (decision date & number: October 16th, 2019/149) in accordance with the Declaration of Helsinki. All volunteers were briefed about the study, and informed consent was obtained.

### Subjects

We referenced the EP 7A-2 protocol of CLSI in the preparation of the plasma pool (*12*). Blood samples were collected into two tubes (BD Barricor lithium heparin plasma tube, Becton, Dickinson and Company, Franklin Lakes, USA) from 20 volunteers who visited our outpatient clinics.

### Methods

Two samples were collected from each volunteer. One of the paired tubes was drawn through a needle (13 mm, 26 gauges) 10 times to obtain a haemolysed specimen. The mechanical haemolysis technique was chosen to produce samples similar to those accepted in the laboratory. No process was performed on the other tube. To avoid possible interference from the matrix, one sample from the same patient was used to form a sample pool without haemolysis, while the other sample was used to form a sample pool with haemolysis. All tubes were centrifuged at 20 °C for 10 minutes at 2000×g according to the manufacturer’s instructions. Tubes were centrifuged within two hours of blood collection. After centrifugation, non-haemolysed samples and haemolysed samples were mixed among themselves. Thence, a non-haemolysed pool and a haemolysed pool were obtained.

The non-haemolysed pool and the haemolysed pool were measured in a blood cell counter (Mindray BC-6800, Shenzhen Mindray Bio-Medical Electronics Co., Ltd, imprecision of Hb ≤ 1.0%) to analyse the free haemoglobin (Hb) values of these samples. First, the specimen containing 10 g/L free Hb was created by diluting the non-haemolysed and haemolysed pools. Subsequently, the haemolysed pools were formed at the adjusted free Hb concentrations of 5 g/L, 2 g/L, and 1 g/L by dilution with the non-haemolysed pool. Five groups with different free Hb concentrations (0 g/L, 1 g/L, 2 g/L, 5 g/L, and 10 g/L) were obtained after these processes.

**Figure 1:**
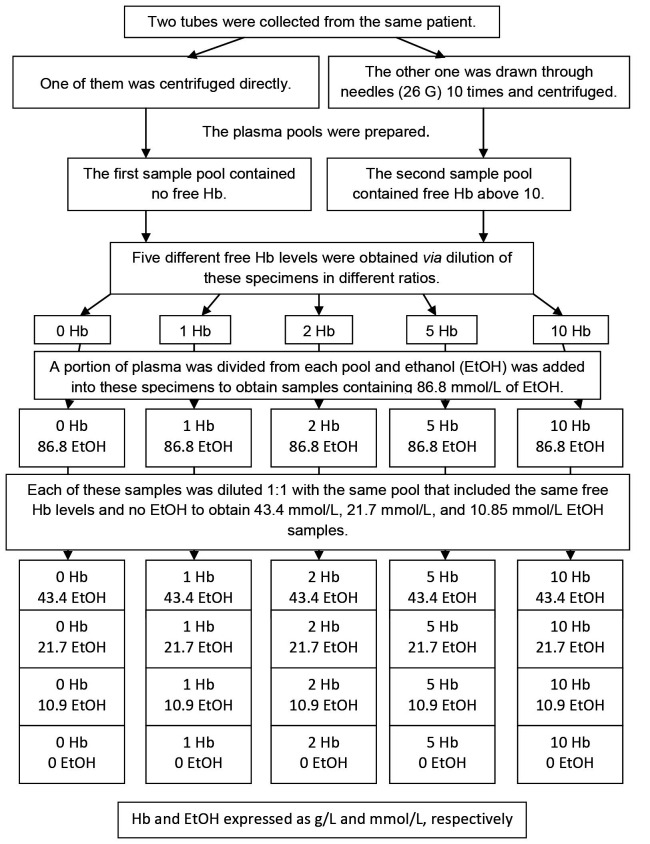
Study design. Preparation process of the sample pools. Hb – haemoglobin. EtOH – ethanol.

The ethanol concentration of each pool was measured and confirmed to contain an ethanol concentration below 1.1 mmol/L. A portion of the plasma was divided from each pool. Ethanol was added to each of these specimens to form samples containing 86.8 mmol/L ethanol. After that, each sample containing 86.8 mmol/L ethanol was diluted at a 1:1 ratio with samples from the same pool at the same free Hb concentrations and without ethanol to avoid dilution bias. The samples containing 43.4 mmol/L, 21.7 mmol/L, and 10.9 mmol/L ethanol concentrations were formed with these procedures (Ethanol absolute EMPLURA, Merck, Burlington, USA). Thus, twenty-five groups were formed with different concentrations of ethanol and free Hb. The design of the study is presented in Figure 1.

Each of the formed groups was divided into 20 equal portions, and an ethanol test was run on each sample. The number of samples *per* group was calculated according to the CLSI EP 7A-2 protocol (*12*). As ethanol is likely to be affected by temperature and humidity, an ethanol test was carried out immediately after each group was formed. During the study, ethanol-containing and non-ethanol-containing samples were kept at 2–8 °C in a closed container. All the measurements have been performed within 2 hours. The ethanol test was carried out with an ethanol assay kit (ETOH, ref no: 474947, Synchron Systems Inc.) using an AU680 autoanalyser (Beckman Coulter, Brea, USA). The principle of the ethanol test is that ADH catalyses the oxidation of ethanol to acetaldehyde with the concurrent reduction of NAD^+^ to NADH. The system monitors the rate of change in absorbance due to NADH at 340 nm.

The Synchron technical report has informed that the assay method for the determination of ethanol provides an analytical measurement range of 1.1-130 mmol/L. It has been declared that the assay ensures a lower limit of quantification of 0.87 mmol/L and a precision between 1.3% and 2.6%. Besides, it is stated that the interference will be 6% in samples with free Hb up to 5 g/L.

### Statistical analysis

The potential effects of haemolysis on ethanol test results were evaluated according to the total allowable error (TEa) recommendation of the Clinical Laboratory Improvement Amendments (CLIA 88) (*13*). It is expected that the calculated TE for the ethanol test will be lower than reported. Another assessment was performed with a maximum acceptable bias of ± 10% for ethanol analysis (*9*, *10*, *14*).

The total error (TE) is calculated as shown below (*15*).

TE = BIAS% + 2CV,

where TE is total error and CV is coefficient of variation

Bias was calculated as follows:


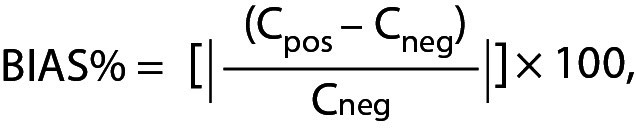


where Cpos represents mean concentration of the haemolysis-positive group and Cneg is mean concentration of the haemolysis-negative group.

## Results

**Figure 2:**
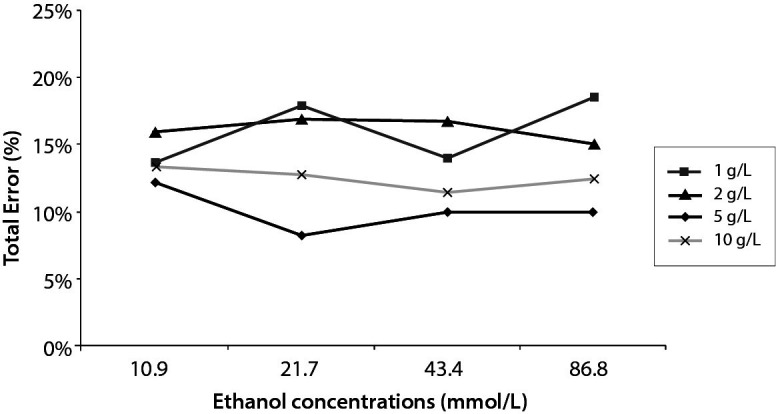
The calculated total errors of the groups are plotted on the graph. Different ethanol concentrations are shown on the x-axis, and the y-axis indicates the percentage of total error.

**Figure 3:**
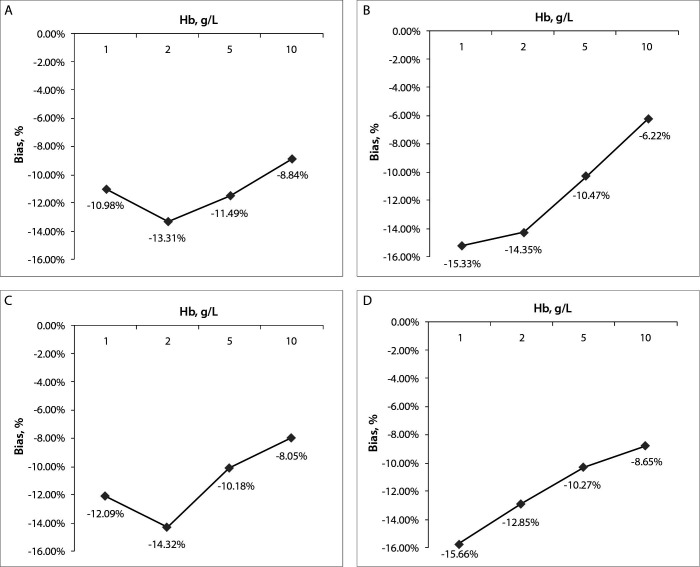
The biases of the groups are represented on the interferograms. Groups were defined according to the ethanol concentrations. A: 10.9 mmol/L, B: 21.7 mmol/L, C: 43.4 mmol/L, D: 86.8 mmol/L.

The TE values in non-haemolysed samples were between 2.04% and 5.06%. The TE values in haemolysed samples were found between 10.12% and 18.66% (Table 1, Figure 2). Haemoglobin-induced bias on ethanol measurement ranged from - 6.22% to - 15.66% (Table 1, Figure 3). According to the obtained data, the maximum acceptable bias was exceeded in samples containing 1, 2 and 5 g/L Hb. The biases in these samples were found to be inconsistent with the manufacturer’s declaration. The mean, CV%, bias%, and TE% values of the groups are shown in Table 1. The TE% and bias% values calculated for each of the evaluated groups are represented in Figure 2 and 3, respectively.

**Table 1:** Mean, CV%, Bias%, and TE% values of the evaluated groups

	**Ethanol 10.85 mmol/L**				**Ethanol 21.7 mmol/L**				**Ethanol 43.4 mmol/L**				**Ethanol 86.8 mmol/L**			
**Hb** **(g/L)**	**Mean**	**CV%**	**Bias%**	**TE%**	**Mean**	**CV%**	**Bias%**	**TE%**	**Mean**	**CV%**	**Bias%**	**TE%**	**Mean**	**CV%**	**Bias%**	**TE%**
0	11.03	1.68	1.70	5.06	21.87	1.69	0.80	4.19	43.66	0.71	0.61	2.04	86.28	1.00	- 0.59	2.58
1	9.66	1.34	- 10.98	13.66	18.37	1.27	- 15.33	17.86	38.15	1.03	- 12.09	14.15	73.21	1.50	- 15.66	18.66
2	9.41	1.36	- 13.31	16.04	18.59	1.29	- 14.35	16.93	37.19	1.18	- 14.32	16.68	75.65	1.03	- 12.85	14.92
5	9.60	0.95	- 11.49	13.39	19.43	1.18	- 10.47	12.82	38.98	0.64	- 10.18	11.46	77.89	1.13	- 10.27	12.53
10	9.89	1.73	- 8.84	12.29	20.35	1.03	- 6.22	8.27	39.90	1.02	- 8.05	10.10	79.29	0.74	- 8.65	10.12
CV – coefficient of variation. TE – total allowable error.																

## Discussion

We observed that haemolysis caused a negative interference for all ethanol concentrations measured from plasma pools with 1, 2, 5 and 10 g/L Hb concentrations. We did not observe a linear relationship between free Hb concentrations and bias/TE% values. Based on the CLIA’88 criteria of the 25% TEa value for ethanol, the TE values at all ethanol concentrations did not exceed the CLIA recommendation.

Gadsden compared the enzymatic method and gas chromatography for ethanol measurement in haemolysed samples. The study showed a significant reduction in ethanol concentration when the haemoglobin concentration was ≥ 5 g/L and a more significant decrease in low ethanol concentration (*8*). Nine *et al.* evaluated the effect of haemolysis in three different enzymatic measurement methods and reported that samples with Hb concentrations between 21.67 and 86.67 g/L did not cause false-positive ethanol results (*6*). Ji *et al.* measured ethanol concentrations in haemolysed plasma on a Roche Cobas 6000 autoanalyser and observed a significant decrease in ethanol concentration at Hb ≥ 2 g/L (*9*). Lippi *et al.* suggested that samples with free Hb concentrations above 38 g/L should be rejected for ethanol measurement (*10*).

All these study results support our findings. However, the mechanism of interference on ethanol testing is not apparent. Gadsden and Ji *et al.* stated in their study that the reason for the interference was spectrophotometric (*8*, *9*). Nine *et al*. evaluated whether lactate dehydrogenase or lactate causes false-positive ethanol results and reported that such an effect might occur according to assay type (*6*). Lippi *et al.* investigated the metabolic aspect of the haemolysis interference and stated that aldehyde dehydrogenase (ALDH) and catalase, which are involved in ethanol metabolism with ADH, may be influential (*10*). The high concentrations of ALDH and catalase released from the lysed erythrocytes are likely to affect the *in vitro* ethanol metabolism (*16*). Since all these enzymes change the concentrations of NAD and NADH, they may affect the enzymatic ethanol measurement. Measurement methods using the absorbance properties of NADPH or NADH (340 nm) are affected by haemolysis (*17*). Spectral interference alone is thought to be close to linear and related to free Hb concentration (*18*). Our findings do not present a linear influence. As shown in other studies, our results were thought to be due to spectral interactions as well as chemical interactions. When all these and our studies outcomes are considered together, we hypothesis that haemolysis causes interference on ethanol measurement with both spectral and chemical mechanisms.

Ethanol is metabolised in the body and excreted from the kidney. Blood ethanol concentration (BEC) decreases in proportion to the time elapsed after drinking an alcoholic beverage. The current sample needs to be analysed because the BEC will have changed in the new sample. Therefore, the rejection criteria of samples accepted into the laboratory for ethanol measurement should be quite rigorous. We think that it may be useful to consider the haemolysis interference when determining these criteria.

Ustundağ *et al.* stated that a TEa value of 25% for ethanol measurement may be high for forensic or clinical critical values (*19*). The recommendation of the Scientific Working Group for Forensic Toxicology regarding the maximum acceptable bias in ethanol measurement is ± 10% (*14*). There is little information for analytical quality specifications of ethanol measurement in the literature. In the current approach, biological variation (BV) is one of the leading options among analytical performance specifications (APS) (*20*). It is stated that BV databases and reference change value (RCV) can be used in haemolysis interference studies (*11*, *21*, *22*). However, since ethanol is not a substance produced in the body, it does not seem to be included in BV studies. The authors could not find any data on ethanol in current BV databases. For this reason, the authors chose to use the value of CLIA recommendation as to the TEa. Although TE approach is a controversial subject in the literature, it has been reported that TE is one of the current options among APS (*23*, *24*). Since the authors chose to use CLIA’s recommendation and the TE approach, they evaluated and presented significant interference in this way. The quality goals for ethanol measurement may be revised accordingly in further studies to determine the bias, CV, and TEa values.

Working Group for Preanalytical Phase (WG-PRE) of European Federation of Clinical Chemistry and Laboratory Medicine has called manufacturers to be more explanatory for the effective use and harmonization of serum indices (*25*). It considers that IVD manufacturers do not precisely comply with CLSI guidelines when performing interference studies (*26*). Therefore, it recommends that each laboratory should perform verification studies on the given interference values. It was emphasized that these indices, which concern both the preanalytical phase and the analytical phase, should be clearly reported in the kit inserts. On the insert sheet of the ethanol kit we evaluated, the manufacturer reported the possibility of interference not exceeding 6% in samples containing 5 g/L Hb. However, our findings were incompatible with this information. In other studies, it has been reported that the values given by the manufacturer are not compatible with their results (*27*, *28*). On the other hand, interferograms are recommended in clinical chemistry tests for managing haemolysed specimens (*11*, *21*). Therefore, it may be more beneficial for manufacturers to document that they have carried out their interference studies following CLSI guidelines and to declare information that can be adapted to interferograms rather than providing single cut-off values.

There are some limitations in our study. Haemoglobin measurement is performed with an automated haematological analyser. The reference method for Hb measurement is spectrophotometric cyanmethemoglobin. It has been reported that the device and method we used in our study is satisfactory for determining the Hb value (*29*, *30*). Additionally, CLSI recommends the use of tubes containing sodium fluoride for the BEC measurement (*31*). Both the kit manufacturer and the tube manufacturer declared that the plasma tubes in our routine usage are suitable for ethanol measurement. The authors desired to design a study that could be applied to the routine functioning of their laboratory.

In conclusion, we demonstrated that ethanol measurement results, carried out by enzymatic methods, may be affected by haemolysis. We observed that negative interference from haemolysis did not exceed the CLIA criteria. Therefore, enzymatic ethanol analysis can be performed on samples containing free Hb up to 10 g/L. Besides, our results are inconsistent with the manufacturer’s statement about haemolysis interference. It may be beneficial for laboratories to have manufacturers carry out interference studies following the guidelines and present results more clearly.
